# Draft Genome Sequence of *Streptococcus thermophilus* C106, a Dairy Isolate from an Artisanal Cheese Produced in the Countryside of Ireland

**DOI:** 10.1128/genomeA.01377-15

**Published:** 2015-11-25

**Authors:** Michiel Wels, L. Mariela Serrano, Beerd-Jan Eibrink, Lennart Backus, Roger S. Bongers, Bastienne Vriesendorp, Roland J. Siezen, Sacha A. F. T. van Hijum, Wilco C. Meijer

**Affiliations:** a CSK food enrichment C.V., Ede, The Netherlands; b NIZO food research B.V., Ede, The Netherlands; c Centre for Molecular and Biomolecular Informatics, Radboud Institute for Molecular Life Sciences, Radboudumc, Nijmegen, The Netherlands; d Microbial Bioinformatics, Ede, The Netherlands

## Abstract

The lactic acid bacterium *Streptococcus thermophilus* is widely used for the fermentation of dairy products. Here, we present the draft genome sequence of *S. thermophilus* C106 isolated from an artisanal cheese produced in the countryside of Ireland.

## GENOME ANNOUNCEMENT

*Streptococcus thermophilus* is a Gram-positive bacterium that is most commonly used in dairy fermentations. It plays an important role in the development of texture and flavor by producing exopolysaccharides and aromatic compounds, but also in food preservation functionality by the production of organic acids (1).

*S. thermophilus* C106 is industrially applied as a starter culture and was isolated from an artisanal cheese produced in the countryside of Ireland. Its pure culture was confirmed to belong to the *S. thermophilus* family by 16sRNA sequencing (data not shown). *S. thermophilus* C106 was grown overnight at 37°C in 100 mL of sterile milk (15 min at 121°C). Milk residues were removed by centrifugation (15 min at 8,000 rpm) after mixing the bacterial culture with 2% Na-citrate in a volume ratio 1:5. Cell pellets were resuspended in a buffer containing 6.7% sucrose, 1 mM EDTA, 50 mMTris-HCl [pH 8.0] and incubated with RNAse (0.5 mg/ml) and lysozyme (2 mg/ml) at 37°C for 1 h. Subsequently, the samples were treated with SDS (1% final concentration) at 37°C for 10 min. The genomic DNA was extracted from the lysate with several phenol/chloroform extractions and precipitated with isopropanol. The DNA was dissolved in sterile water.

Whole-genome sequencing was performed at GATC biotech (Konstanz, Germany*)*. The libraries were paired-end 50 bp and sequenced using an Illumina HiSeq 2000. Raw sequence reads were assembled *de novo* using the CLC Genomics Workbench using standard settings (http://www.clcbio.com). The *S. thermophilus* C106 genome was assembled into 87 contigs and is estimated to be 1.77 Mb with a G+C content of 38.95%.

Annotation was performed by automated annotation of the contig sequences by the RAST server (2). In total 1,945 open reading frames were predicted.

### Nucleotide sequence accession numbers.

This whole-genome shotgun project has been deposited at DDBJ/EMBL/GenBank under the accession LGRS00000000. The version described in this paper is version LGRS01000000.
